# Treatment of a Maxillary Second Molar with One Buccal and Two Palatal Roots Confirmed with Cone-Beam Computed Tomography 

**DOI:** 10.22037/iej.v12i3.16331

**Published:** 2017

**Authors:** Masoud Parirokh, Mahsa Razifar, Hamed Manochehrifar, Paul V Abbott, Nima Hatami, Nargessaddat Kashi, Aida Farhadi

**Affiliations:** a *Endodontology Research Center, Kerman University of Medical Sciences, Kerman, Iran*;; b *Endodontic Department, Dental School, Kerman University of Medical Sciences, Kerman, Iran;*; c *Oral and Dental Diseases Research Center, Kerman University of Medical Sciences, Kerman, Iran*;; d Dental *School, University of Western Australia, Perth, Australia*

**Keywords:** Cone-Beam Computed Tomography, Maxillary Second Molar, Palatal Roots, Root Anatomy

## Abstract

Root canal configuration is an important subject in endodontic practice and dentists should be familiar with all possible types of root canal configuration. A forty-three year old male was referred for root canal treatment of his maxillary left second molar tooth with symptomatic irreversible pulpitis. Pre-operative radiographs showed a three rooted molar. However, after access cavity preparation two palatal and one buccal orifices were detected. The patient was informed of the unusual root canal anatomy and cone-beam computed tomography (CBCT) was ordered for precise evaluation of the anatomy. CBCT image confirmed the presence of one buccal and two palatal root canals; an exceptionally rare condition.

## Introduction

Patients attend endodontic offices for a variety of reasons from diagnosis of the etiology of their pain to receiving treatment for tooth decay and traumatic injuries [1, 2]. One of the most important parts of endodontic practice is locating and negotiating all root canals and clean them to their optimal working length. This is required in order to clean and shape the canals so all microorganisms and pulp tissue are removed. Thorough attention to this aim will lead to more predictable treatment outcomes [3]. Therefore, all dentists should have a thorough knowledge of all possible variations of root canal morphology in all teeth [4]. 

Numerous investigations have been performed on root canal morphology of various teeth to increase the knowledge of dentists in this regard [5-9]. Similar to their mandibular counterparts, maxillary molars have a variety of root canal morphologies with the reported number of canals ranging from one to seven [10-14]. Most articles regarding the anatomy of maxillary second molars are case reports and case series and these present various configurations of this tooth [15-27]. Most investigations of large numbers of maxillary teeth have reported that maxillary second molars generally have three separate root canals with two in the buccal and one in the palatal roots [5, 6, 28]. Another less common configuration of maxillary second molar is presence of two distinct buccal and palatal roots. Presence of maxillary second molars with four roots including two buccal and two palatal roots is also found with lower percentage compared to the other canal configurations of the tooth [6, 10]. One case report has presented a maxillary second molar with one buccal and one palatal roots where the palatal root had two root canals [23]. 

This case report presents a rare case of a maxillary second molar tooth with three separate roots, two of which were located palatally and the other one was on the buccal aspect of the tooth.

## Case Report

**Figure 1:**
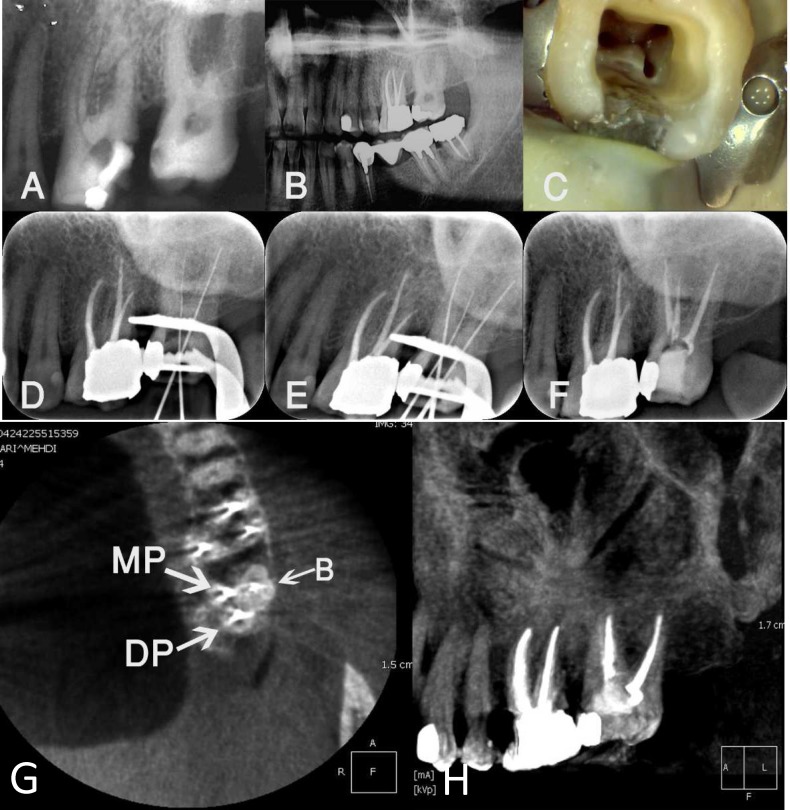
)* Pre-operative periapical radiography of the maxillary left second molar before restoration with amalgam;* B)* Panoramic view of the tooth after amalgam placement and following episodes of pain;* C)* Detecting buccal and distobuccal root canals after file placement and taking radiography;* D) *Root canal orifices of the tooth; one orifice is on the buccal and there are orifices to two palatal root canals;* E)* Radiograph taken after detecting all root canals;* F)* Periapical radiography after completion of the root canal filling;* G)* Axial CBCT view;* H)* Sagittal CBCT view*

A 43 year old male presented with severe sensitivity to cold and spontaneous pain in the left side of his upper jaw. His medical history was not contributory. His dental history showed that the maxillary left second molar had a carious cavity on the mesial surface (Figure 1A) which was restored with an amalgam restoration one year earlier (Figure 1B). However, he recently had experienced episodes of spontaneous severe pain. During clinical examination the patient had lingering pain following the application of a cold pulp sensibility test using a cold spray (Monoart, Euronda, Italy). The tooth had sensitivity to heat and electric pulp tests and mild tenderness on percussion. Therefore, the diagnosis of symptomatic irreversible pulpitis was made. 

After administration of local anesthesia using 2% lidocaine with 1:80000 epinephrine (Lidocaine, DarouPakhsh, Tehran, Iran), rubber dam was placed and access cavity preparation was commenced. After removing the roof of the pulp chamber and the coronal pulp, a DG-16 explorer (Hu-Friedy, Chicago, IL, USA) was used to locate the root canal orifices. However, in contrast with what was expected, only one buccal and one palatal orifice were initially detected. A periapical radiography was taken which showed that the second canal was not located in the mesial aspect of the tooth (Figure 1C). After careful exploration of the floor of the pulp chamber, another orifice was detected on the mesio-palatal aspect of the access cavity (Figure 1D). The patient was informed about the unusual root canal anatomy of this tooth and the possibility that there may even be more canals. The clinician recommended that the root canal anatomy should be evaluated with three-dimensional cone-beam computed tomography (CBCT). However, the patient declined this and said he would rather finish the root canal treatment at that visit.

After negotiating the root canal length with a #10 K-file (Dentsply Maillefer, Ballaigues, Switzerland), the coronal two thirds of the root canals were enlarged with #2 and 3 Gates Glidden drills (Dentsply Maillefer, Ballaigues, Switzerland). After estimating the canal lengths with an electronic apex locator (Root ZX, J. Morita, Tokyo, Japan) and confirming the working lengths with a periapical radiography (Figure 1E), the canals were prepared with RaCe rotary instruments (FKG Dentaire, La-Chaux-de Fonds, Switzerland) in a crown down manner. The master apical file for the palatal root canals was set as 25/0.06, while size 30/0.06 was selected as the master apical file for the single buccal canal. Between each instrument the canals were irrigated with 5.25% NaOCl. After instrumentation, the canals were irrigated with ethylene diamine tetra acetic acid (EDTA) (Asia Chimi Teb, Tehran, Iran) to remove the smear layer, followed by a copious amount of 0.9% normal saline. The root canals were then dried with paper points and filled with lateral condensation of gutta-percha (Meta Biomed co., Chungcheongbuk do, Korean) and AH-26 root canal sealer (Dentsply, DeTrey, Konstanz, Germany). A sterile cotton pellet was placed in the pulp chamber and the tooth was temporarily restored (MD Temp, Meta Biomed, Chungcheongbuk do, Korea) (Figure 1F).

One week later a CBCT image was taken before coronal restoration in order to have further information regarding the number and configuration of the root canals and to ensure that no canals were missed. The CBCT image was evaluated by a map reading method to interpret all axial, coronal and sagittal images to be sure that all root canals that might be present on the buccal and palatal sides of the tooth were identified. The CBCT images revealed one large root on the buccal, and two separate roots on the palatal of the tooth (Figures 1G and H). Only one canal was found in each root and each of these was filled with radiopaque materials.

## Discussion

This case report presents an unusual case of a maxillary second molar with one buccal and two palatal roots. Awareness of root canal anatomy and possible canal abnormalities is essential for successful root canal treatment [29]. 

CBCT is a helpful device for evaluation of root canal anomalies and it can also be used for intra- or post-operative assessment of treatment complications. Use of CBCT imaging could minimize unnecessary extension of access cavities and excess dentine removal while searching for and negotiating root canal orifices [29, 30]. One of the CBCT applications is evaluation of root canal configuration [31]. In the present case, the patient had been informed about the unusual anatomy of his tooth and he was advised to have a CBCT image taken to be sure about the root canal anatomy. Every canal that might have been missed could be located during treatment.

In accordance with the recommendations of the American Associations of Endodontists (AAE) and the American Academy of Oral and Maxillofacial Radiology (AAOMR) [32] as well as European Society of Endodontology (ESE) [33], CBCT should be considered as an adjunct to two-dimensional imaging in dentistry only in necessary situations where low-dose conventional dental radiography or alternate imaging modalities are not adequate enough [32]. The CBCT image was considered after finishing the treatment because the patient initially preferred to complete the treatment without CBCT 

evaluation. However, he subsequently changed his mind and took the CBCT before application of the final restoration. It would have been more desirable to take the CBCT image before the root canal treatment was completed because of the potential artifact effect of the filling material which may mask the presence of untreated missed root canals [29, 34]. If the image had been taken prior to completing the root canal treatment, then any further canals identified by the CBCT image could have been more easily treated.

CBCT is known to be a very useful method for evaluating root canal anatomy. Several investigations have evaluated root canal morphology of maxillary molars. Zhang *et al.* [28] clarified the effectiveness of CBCT in detection of root canals and identification of their configurations. They also concluded that maxillary second molars have more complicated root canal anatomy than maxillary first molars. 

One of the main concerns of professional associations and societies such as the AAE and ESE has been the routine ordering of CBCT imaging by dental practitioners prior to any dental procedure [32, 33]. Both the AAE and ESE in their position statements have emphasized that CBCT should only be ordered if one or several important reasons are present to justify a clinician’s decision and based on cost-effectiveness for the patient. In addition, the ESE has emphasized that dentists who are using or ordering CBCT images should have adequate education and training in the interpretation of the data set provided by this technique. The European Academy of DentoMaxilloFacial Radiology has announced basic training requirements for dentists who are going to use CBCT in their daily practice [35]. One of the indications for ordering CBCT is to investigate root canal morphology. In the present case, CBCT help the clinicians to be sure of the number of roots. It was also useful to evaluate the technical aspects of the root canal filling [29].

Some authors believe that a palato-gingival groove may be formed as a result of two palatal roots in maxillary molars [36]. However, in this case, no palate-gingival groove was observed. The incidence of maxillary second molars with four roots, including two palatal roots, has been reported to be 0.4% [8]. However, none of the recent studies of maxillary second molars using CBCT imaging report any cases with two distinct palatal roots [5-7, 28].

Christie *et al.* [16] introduced a classification system for four-rooted maxillary second molar abnormalities based on the tooth root separation level and divergence. However, the current case did not match any of the described categories in their classification system as this system only includes teeth with four roots.

To the authors’ knowledge, this is the first time a maxillary second molar with one buccal and two palatal canals has been reported. The difficulty of locating this type of canal abnormality should be considered because of the posterior location of the tooth and difficult access as well as the superimposition of anatomic structures on plain radiographs.

## Conclusion

Although very rare, the presence of two palatal roots and one buccal canal should be anticipated as a possible occurrence when providing root canal treatment for maxillary second molars.
